# Evaluation of QuantiFERON-TB Gold Plus for Predicting Incident Tuberculosis among Recent Contacts: A Prospective Cohort Study

**DOI:** 10.1513/AnnalsATS.201905-407RL

**Published:** 2020-05

**Authors:** Rishi K. Gupta, Heinke Kunst, Marc Lipman, Mahdad Noursadeghi, Charlotte Jackson, Jo Southern, Ambreen Imran, Stefan Lozewicz, Ibrahim Abubakar

**Affiliations:** ^1^ University College London London, United Kingdom; ^2^ Queen Mary University of London London, United Kingdom; ^3^ Royal Free London NHS Foundation Trust London, United Kingdom; ^4^ Public Health England Colindale London, United Kingdom and; ^5^ North Middlesex University Hospital NHS Trust London, United Kingdom

*To the Editor*: Screening for latent tuberculosis infection (LTBI) among recent tuberculosis (TB) contacts is an important component of TB control, particularly in settings with low TB incidence aiming toward pre-elimination (1). However, currently available LTBI diagnostics lack sensitivity and have poor predictive value for incident TB (2–8). Consequently, prevention of one incident TB case requires treatment of many individuals for LTBI. This is true for both interferon-γ release assays (IGRAs) and the tuberculin skin test (TST). A recent evaluation found that QuantiFERON Gold-In-Tube (QFT-GIT; Qiagen) and T-SPOT.TB (Oxford Immunotec) performed similarly to the TST when a Bacillus Calmette–Guerin vaccination-stratified TST cutoff was used (8).

 A new-generation QuantiFERON (QuantiFERON-TB Gold Plus; QFT-Plus) was recently launched, adding a second TB antigen tube (TB2) that incorporates short peptides designed to stimulate a CD8^+^ T-cell response, in addition to the CD4^+^-response tube (TB1) included in previous versions. The proposed rationale for this is that CD8^+^ responses have been associated with mycobacterial load and recent TB exposure (9, 10). Initial independent evaluations have suggested that QFT-Plus may have improved test sensitivity in active TB compared with QFT-GIT (11), and that the CD8^+^-targeted antigen tube response may be associated with proxy measures of the degree of TB exposure among contacts (12). However, no studies have examined the prognostic value of QFT-Plus for predicting incident TB. We aimed to address this key knowledge gap in a prospective cohort of TB contacts in the United Kingdom.

## Methods

We recruited adult (≥16 yr old) contacts of pulmonary and extrapulmonary TB index cases from 10 London TB clinics while attending for routine contact screening (July 7, 2015 to November 22, 2016). Participants completed a questionnaire and underwent blood sampling for QFT-Plus (at least 6 weeks from the last known TB exposure). Contacts with evidence of prevalent TB disease (defined as TB diagnosed within 21 days of enrollment, as per previous work [8]) and those who accepted preventive therapy (offered in accordance with contemporary national guidance [13, 14]) were excluded from the analysis. The study was approved by the UK National Research Ethics Service (ref: 14/EM/1208).

Participants were linked to national TB surveillance records held by Public Health England, including all statutory TB notifications, to identify those notified with TB (until December 31, 2017). TB notifications were validated by local record review and included those with culture-confirmed TB or a clinical diagnosis with radiological or histological evidence of TB, for which a clinician had prescribed a full course of anti-TB treatment.

The QFT-Plus results were interpreted according to the manufacturer’s guidance, with TB antigen responses calculated as TB antigen interferon-γ minus unstimulated control interferon-γ. We calculated incidence rates and rate ratios (IRRs) relative to the negative test category, along with sensitivity, specificity, and predictive values, including the full duration of follow-up.

To assess the incremental value of adding the CD8^+^-stimulating tube in predicting incident TB cases, we compared receiver operating characteristic (ROC) curves and area under the curve (AUC) values obtained using TB1 only, TB2 only, and the maximal TB antigen tube (higher of TB1 and TB2). We also plotted a ROC curve for the calculated difference between the TB1 and TB2 tubes (TB2 − TB1) as a surrogate for the CD8^+^-specific response, as it has been hypothesized that this may identify contacts with recently acquired *Mycobacterium tuberculosis* infection, who are at the highest risk of TB disease (12).

## Results

We recruited a total of 623 contacts, 532 (85.4%) of whom had QFT-Plus results (89 missing and 2 indeterminate) and were followed for a median 1.93 years (interquartile range [IQR] 1.65–2.21 yr). QFT-Plus results were positive in 180 of the 532 contacts (33.8%) (Table 1), and 39 (21.7%) of these participants commenced preventive therapy. One patient was notified with prevalent TB (3 days after recruitment). A total of 492 participants were therefore included in the analysis. The included and excluded participants had similar baseline characteristics, except that those who commenced preventive therapy were younger than those who did not (Table 1).

**Table 1.** Baseline characteristics of the study cohort, stratified by Quantiferon-TB Gold Plus results and provision of preventive therapy

	QFT-Plus Negative*	QFT-Plus Positive		QFT-Plus Missing^†^	All
		No PT*	PT		
Age					
Median (IQR)	31 (25–43)	43 (32–54)	30 (26–35)	31.5 (23.7–49)	33 (25–46)
Missing	3 (0.9)	2 (1.4)	0 (0)	1 (1.1)	6 (1)
					
Sex					
Male	165 (46.9)	76 (53.9)	24 (61.5)	37 (40.7)	302 (48.5)
Female	180 (51.1)	62 (44)	15 (38.5)	51 (56)	308 (49.4)
Missing	7 (2)	3 (2.1)	0 (0)	3 (3.3)	13 (2.1)
					
Ethnicity					
White	95 (27)	27 (19.1)	9 (23.1)	31 (34.1)	162 (26)
South Asian	117 (33.2)	55 (39)	13 (33.3)	33 (36.3)	218 (35)
Black African or Caribbean	67 (19)	30 (21.3)	7 (17.9)	15 (16.5)	119 (19.1)
Other	63 (17.9)	24 (17)	10 (25.6)	9 (9.9)	106 (17)
Missing	10 (2.8)	5 (3.5)	0 (0)	3 (3.3)	18 (2.9)
					
UK born					
No	235 (66.8)	126 (89.4)	33 (84.6)	66 (72.5)	460 (73.8)
Yes	111 (31.5)	11 (7.8)	6 (15.4)	24 (26.4)	152 (24.4)
Missing	6 (1.7)	4 (2.8)	0 (0)	1 (1.1)	11 (1.8)
					
Contact type					
Household	210 (59.7)	96 (68.1)	30 (76.9)	49 (53.8)	385 (61.8)
Family nonhousehold	19 (5.4)	7 (5)	2 (5.1)	3 (3.3)	31 (5)
Work or social	62 (17.6)	19 (13.5)	4 (10.3)	14 (15.4)	99 (15.9)
Other	13 (3.7)	3 (2.1)	2 (5.1)	2 (2.2)	20 (3.2)
Missing	48 (13.6)	16 (11.3)	1 (2.6)	23 (25.3)	88 (14.1)
					
Diabetes					
No	318 (90.3)	120 (85.1)	38 (97.4)	83 (91.2)	559 (89.7)
Yes	20 (5.7)	18 (12.8)	0 (0)	6 (6.6)	44 (7.1)
Missing	14 (4)	3 (2.1)	1 (2.6)	2 (2.2)	20 (3.2)
					
HIV					
No	331 (94)	137 (97.2)	37 (94.9)	84 (92.3)	589 (94.5)
Yes	4 (1.1)	0 (0)	1 (2.6)	2 (2.2)	7 (1.1)
Missing	17 (4.8)	4 (2.8)	1 (2.6)	5 (5.5)	27 (4.3)
					
Follow-up, yr					
Median (IQR)	1.94 (1.64–2.21)	1.92 (1.66–2.21)	1.85 (1.67–2.25)	1.56 (1.25– 2.06)	1.88 (1.58–2.20)
					
Total	352	141	39	91	623

*Definition of abbreviations*: HIV = human immunodeficiency virus; IQR = interquartile range; PT = preventive therapy; QFT-Plus = QuantiFERON-TB Gold Plus.

Data are presented as *n* (%) unless stated otherwise.

* Included in the primary analysis.

^†^ Includes two patients with indeterminate QFT-Plus results.

Ten patients with incident TB were notified during follow-up (median 222 days after recruitment; range 90–688). Among these patients, the median age was 27 (IQR 21–33), three (30%) were female, the majority (7/10; 70%) were of black African or South Asian ethnicity, and all were non-UK born. All 10 patients completed at least 6 months of TB therapy. Two of these cases (20.0%) were pulmonary in site (both were culture confirmed), and eight were exclusively extrapulmonary (two of which were culture confirmed). One participant with TB was diabetic; the remaining patients with TB were not immunocompromised, and none were infected with human immunodeficiency virus. The TB incidence rates (per 1,000 person-years) were 30.6 (95% confidence interval [CI], 15.3–61.1) and 3.0 (95% CI, 0.8–12.1) in the QFT-Plus–positive and –negative groups, respectively (IRR, 10.1 [95% CI, 2.2–47.7]). The sensitivity of QFT-Plus for incident TB was 80.0% (95% CI, 44.4–97.5). The positive predictive value (PPV) and negative predictive value were 5.7% (95% CI, 2.5–10.9) and 99.4% (95% CI, 98.0–99.9), respectively. The characteristics of QFT-Plus for predicting microbiologically confirmed TB cases are reported in Table 2.

**Table 2.** Incidence rates, rate ratios, and predictive values for incident tuberculosis during follow-up, stratified by Quantiferon-TB Gold Plus results

	QFT-Plus Positive	QFT-Plus Negative
No. of TB cases (microbiologically confirmed and/or clinically diagnosed)	8	2
Participants	140	352
Person-years	261.6	663.0
Incidence rate per 1,000 person-years	30.6 (15.3–61.1)	3.0 (0.8–12.1)
Incidence rate ratio	10.1 (2.2–47.7)	
Positive predictive value	5.7 (2.5–10.9)	
Negative predictive value	99.4 (98–99.9)	
Sensitivity	80.0 (44.4–97.5)	
Specificity	72.6 (68.4–76.5)	
	
No. of TB cases (microbiologically confirmed only)	3	1
Incidence rate per 1,000 person-years	11.5 (3.7–35.6)	1.5 (0.2–10.7)
Incidence rate ratio	7.6 (0.8–73.1)	
Positive predictive value	2.1 (0.4–6.1)	
Negative predictive value	99.7 (98.4–100)	
Sensitivity	75.0 (19.4–99.4)	
Specificity	71.9 (67.7–75.9)	

*Definition of abbreviations*: QFT-Plus = QuantiFERON-TB Gold Plus; TB = tuberculosis.

**Figure 1.**
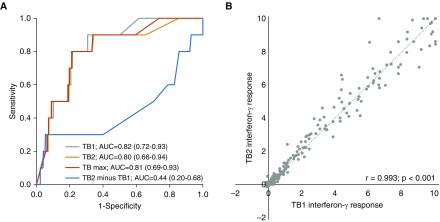
(*A*) Receiver operating characteristic curves showing the performance of QuantiFERON-TB Gold Plus for predicting incident tuberculosis during the duration of follow-up, stratified by antigen tube interferon-γ responses. TB max = higher of the CD4^+^-response tube (TB1) and CD8^+^-response tube (TB2). AUC = area under the curve (95% confidence interval). (*B*) Scatterplot showing association of interferon-γ responses in the TB1 and TB2 tubes.

ROC curves for prediction of incident TB during all follow-up were similar for the TB1, TB2, and maximal TB antigen responses (AUC 0.80–0.82; Figure 1A). TB2 minus TB1, however, did not discriminate TB progressors from nonprogressors (AUC 0.44 [95% CI, 0.20–0.68]). There was a very strong correlation between the TB1 and TB2 interferon-γ responses (*r* = 0.993; *P* < 0.001; Figure 1B).

## Discussion

We found that the performance of QFT-Plus appeared to be comparable to that previously reported in evaluations of QFT-GIT and T-SPOT.TB, with an IRR of 10.1, 80% sensitivity for detection of incident TB, and an overall PPV for incident TB of 5.7% (8). Interferon-γ responses in the TB1 and TB2 tubes were strongly correlated, and ROC curves showed a minimal difference between them for predicting incident TB. As a result, the calculated difference between TB1 and TB2 responses, as a proxy for the CD8-specific response, did not predict incident TB. However, despite our sample size of 492 recent TB contacts, the number of TB progressors was small, reflecting a low progression risk even among contacts. Thus, a larger-scale study is indicated to investigate subtle differences in the relative prognostic contributions of the TB1 and TB2 antigen tubes.

This is the first evaluation of the prognostic value of the QFT-Plus test. The prospective design allowed the collection of detailed clinical, demographic, and laboratory data. We recruited participants while they were receiving routine contact-tracing services, to ensure that the study population would be representative of TB contacts. Therefore, our findings are likely generalizable to other low-incidence settings globally. Moreover, follow-up was robust through linkage to national surveillance records using a validated matching algorithm (15), minimizing the risk of missing incident TB cases.

A limitation of this study is that the provision of preventive therapy to a subset of the QFT-Plus–positive patients could have led to selection bias. However, although the patients who received preventive therapy were younger than those who did not (reflecting national policy at the time of the study [13, 14]), other characteristics were similar between the two groups, suggesting that the impact of this bias was likely small. Second, the TB contacts included both pulmonary and extrapulmonary index cases, reflecting national contact screening policy during the study period (13). The PPV of QFT-Plus may be higher among populations that include only pulmonary TB contacts, owing to a higher pretest probability of incident TB. However, previous evaluations of QFT-GIT and T-SPOT.TB also included extrapulmonary contacts, which allows the current findings to be put into this context (8). Third, we did not perform serial testing (before and after exposure), so we were unable to assess QFT-Plus conversions over time, which may provide a more reliable measure of recent *M. tuberculosis* infection. This reflects the reality of contact screening practices—the ability of assays to accurately stratify TB risk from a single baseline test is therefore a key attribute. The absence of serial testing also means that participants who developed incident TB may have been reexposed to *M. tuberculosis* during the interval between testing and disease onset, although the overall risk of exposure in the United Kingdom (a low-TB-incidence setting) is likely small. Fourth, QFT-Plus results were missing or indeterminate for 91 of 623 patients (14.6%), in keeping with the proportion of missing results for other IGRAs in our recent evaluation (8). However, these patients’ characteristics were similar to those of the overall study population, suggesting that the risk of subsequent selection bias was likely small. Finally, we included both microbiologically confirmed and clinically diagnosed TB cases in our outcome definition, in keeping with previous IGRA evaluations (3–8). The rationale for this is that extrapulmonary TB, which accounts for a large proportion of TB cases in foreign-born people living in the United Kingdom (16), is often challenging to prove microbiologically. However, all patients who received a diagnosis of TB during the study received a full course of TB therapy, and none were denotified. It is therefore likely that these represented true TB cases, with a low risk of outcome misclassification.

In summary, in this first evaluation of the predictive value of QFT-Plus for incident TB, we found that its performance was comparable to that of other commercial IGRAs. Better biomarkers are required to transform management of TB contacts.

## Supplementary Material

Supplements

Author disclosures
