# Supplementary material for: Evaluation of QuantiFERON-TB Gold Plus for Predicting Incident Tuberculosis among Recent Contacts: A Prospective Cohort Study
Source: Ann Am Thorac Soc. 2020 May;17(5):646–50. doi: 10.1513/AnnalsATS.201905-407RL (PMC7193805; doi:10.1513/AnnalsATS.201905-407RL)
Supplement: Supplements [file AnnalsATS.201905-407RL.html]

Evaluation of QuantiFERON-TB Gold Plus for Predicting Incident Tuberculosis among Recent Contacts: A Prospective Cohort Study | Annals of the American Thoracic Society

- disclosures.pdf (311 KB)
